# Spectroscopic and Theoretical Investigation of Water Binding in a Copper–Calcium Complex

**DOI:** 10.1021/acs.jpca.5c03616

**Published:** 2025-07-24

**Authors:** Noël de Kler, Aleksandr Y. Pereverzev, Jana Roithová

**Affiliations:** Department of Spectroscopy and Catalysis, Institute for Molecules and Materials, 6029 Radboud University, 6525 AJ Nijmegen, The Netherlands

## Abstract

Water oxidation catalysis relies critically on the organization of water molecules near reactive centers. Inspired by the Oxygen Evolving Complex in Photosystem II, we developed a copper–calcium model complex to investigate water coordination effects. We synthesized and characterized a [Cu­(L-H)­(BF_4_)] complex featuring a tetradentate N_3_O ligand. Upon the addition of calcium hydroxide, the complex transforms into a stable copper–calcium complex with two coordinated water molecules. Detailed characterization by ultraviolet–visible (UV–vis) spectroscopy, electrospray ionization mass spectrometry (ESI-MS), helium-tagging IR photodissociation spectroscopy, and density functional theory (DFT) calculations revealed the structure and hydrogen-bonding network within the complex. The data demonstrate that water molecules are preorganized via calcium coordination and hydrogen bonding to the ligand. Such tight coordination of water molecules in the vicinity of the copper reaction center could facilitate selective O–O bond formation in water oxidation processes by stabilizing reactive intermediates and preventing deleterious side reactions.

## Graphical abstract

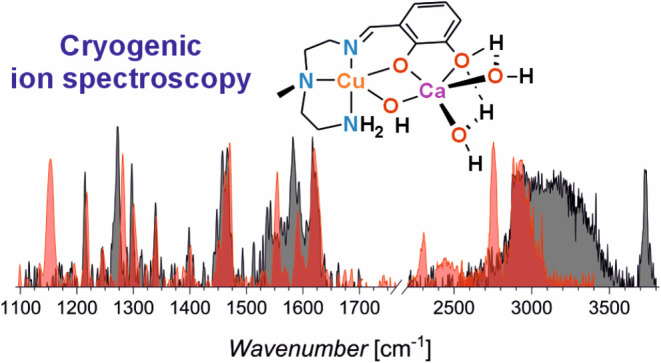

## Introduction

Water splitting is the key reaction in transforming a hydrocarbon-based economy toward more sustainable variants. Much effort is devoted to optimizing catalysts for both sides of the reaction: the hydrogen evolution reaction and the oxygen evolution reaction. The latter is a highly demanding reaction requiring a transfer of four electrons and a system enabling an efficient O–O bond formation. Nature optimized the Oxygen Evolving Complex within photosystem II, containing four manganese and one calcium centers arranged in a metal-oxo cluster shown in Figure 1a.^1,2^ The complex can accumulate high redox potential via sequential electron- and proton-transfer steps, forming a manganese-oxyl intermediate. The oxyl then reacts with a calcium-bound water/hydroxyl to form the O–O bond.^3^ Hence, the manganese metals play an important role in mediating the oxidation chemistry, whereas the calcium center is primarily involved in preorganizing the water molecules to react.^4^

**Figure 1:**
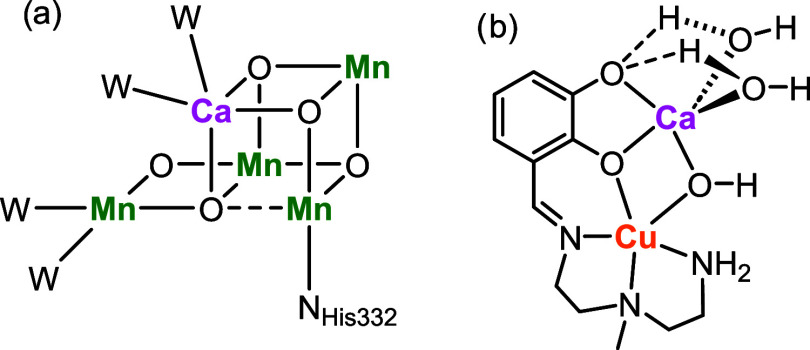
(a) Oxygen Evolving Complex (W = water molecule, N_His332_—binding of a histidine residue of the protein).^1,2^ (b) Copper–calcium complex investigated here.

Extensive work focused on building synthetic and theoretical models to understand the details of the oxygen-evolving complex.^4–12^ The focus was primarily on the oxidation steps toward forming the reactive manganese-oxyl unit. However, the overall reaction is not accomplished after the manganese-oxyl unit is formed; the follow-up reaction with water is important, too. A binding site for the precoordination of the water molecule and a network of hydrogen bonds can tremendously affect the kinetics and, thus, the selectivity of the reaction.^13–16^ However, investigating water molecules’ coordination and hydrogen bonding is challenging. One of the experimental approaches is based on isolating metal complexes with coordinated water molecules in the gas phase, which can be followed by detailed spectroscopic investigation.^17–22^ Here, we present a copper complex offering an additional coordination site to a calcium ion, which binds water molecules and thus forms a stable water-containing coordination sphere of copper (Figure 1b). For the water oxidation reaction, the electronic properties of the ligand must be optimized to achieve optimal performance. Nevertheless, this simple model serves well to investigate the nature of water molecules binding and the effect of hydrogen bonding within the complex.

## Methods

[Cu­(BF_4_)_2_] hexahydrate (202 mg, 0.852 mmol, 1eq) was dissolved in a mixture of methanol/acetonitrile (1:1, 8 mL). A solution of 2,2′-diamino-*N*-methyldiethylamine (110 μL, 0.852 mmol) in 4 mL methanol was slowly added to the copper solution while stirring. After the addition, the solution was stirred for ∼5 min, followed by adding 2,3-dihydroxybenzaldehyde (118 mg, 0.852 mmol) in 2 mL of methanol while stirring. After stirring the solution for 3 h, diethyl ether (∼20 mL) was added, resulting in the precipitation of the complex. The solution was filtered via vacuum filtration. The solids on the filter were washed with diethyl ether and dried to obtain a red crystalline material (170 mg, 52% yield). ESI-MS (H_2_O), *m*/*z*: 299 ([(Cu­(L-H))]^+^), 685 ([(Cu­(L-H))_2_(BF_4_)]^+^). Single crystals were grown from a solution of [(Cu­(L-H)­(BF_4_))] in methanol layered with diethyl ether. Slow diffusion of diethyl in methanol resulted in the formation of red crystals, which were characterized by X-ray diffraction (deposition number 2314714). For more information, see the Supporting Information.

The electrospray ionization mass spectrometry (ESI-MS) experiments were performed with a triple-quadrupole instrument, TSQ 7000 (Thermo), equipped with an electrospray ionization (ESI) source. High-resolution mass spectrometry experiments were performed with a timsToF instrument (Bruker, Germany) with an ESI source. The ions of interest were obtained by soft electrospray ionization from an aqueous solution of [Cu­(L-H)­(BF_4_)] (0.1 mM) and calcium hydroxide (1.33 mM). The ultraviolet–visible (UV–vis) spectra were recorded with a JASCO V630 UV–vis spectrophotometer. For more information, see the Supporting Information.

IRPD experiments were performed with the customized ISORI instrument. The instrument is equipped with an electrospray ionization (ESI) source and has a quadrupole (Q1)—quadrupole bender (QB)—octupole (O)—quadrupole ion trap (QIT)—quadrupole (Q2) geometry. The ions generated by ESI are mass-selected by Q1 and guided by QB and O to the quadrupole ion trap operating at ∼3.5 K. The ions are trapped and thermalized by collisions with helium buffer gas (Scheme S1). Helium is injected by a piezo valve in several 0.2 ms pulses separated by a 20 ms delay for a time of 200 ms. The thermalized ions M^+^ form complexes with helium atoms MHe^+^. The ions are then ejected from the trap, and the number of helium-tagged ions MHe^+^ is determined by mass analysis by Q2 and detected by a dynode multiplier system operated in a counting mode. The experiment works with a 1 Hz frequency, and the trapped ions are irradiated in alternating cycles by a Nd/YAG laser-pumped tunable OPO/OPA system (Laser Vision). The number of helium-tagged ions is *N_i_* and *N*_*i*0_ in the cycle with and without irradiation. The IRPD spectrum is constructed as (1 – *N_i_*/*N*_*i*0_).

The density functional theory calculations were performed with the B3LYP functional using the D3BJ empirical correction for the dispersion interactions and the 6–311++G** basis set. All structures were fully optimized and confirmed by the frequency calculations. The reported theoretical IR spectra are harmonic and scaled by 0.985 below 2000 cm^–1^ and 0.955 above 2000 cm^–1^. The calculations were performed with the Gaussian package.

## Results

### The [Cu­(L-H)­(BF_4_)] Complex

The copper complex with the N_3_O tetradentate ligand L was prepared in one step by mixing triamine **A**, 2,3-dihydroxybenzaldehyde (**B**), and copper­(II) tetrafluoroborate (Figure 1, see the Experimental Details for more information). The triamine and aldehyde condensed to form the tetradentate ligand L. The copper ions coordinated with singly deprotonated ligand L. Recrystallization of the obtained complex yielded single crystals of [Cu­(L-H)­(BF_4_)], which were characterized by X-ray diffraction (Figure 2). The ligand offers a square planar N_3_O coordination site, and the BF_4_^–^ coordinates above the plane.

**Figure 2:**
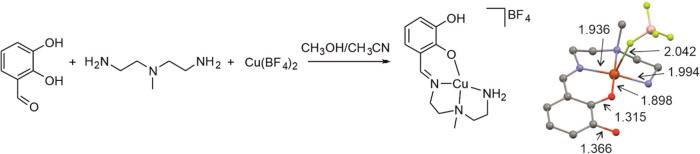
(a) Synthesis of [Cu­(L-H)­(BF_4_)] (see the Experimental Details for details). (b) The crystal structure of [Cu­(L-H)­(BF_4_)]. The distances are given in Å. Color code: Carbon = black, hydrogen = white, oxygen = red, nitrogen = blue, Copper = yellow, boron = pink and fluor = green. The remaining hydrogen atoms are omitted for clarity.

### Binding of the [Cu­(L-H)­(BF_4_)] Complex with Calcium Ions

The UV–vis spectrum of [Cu­(L-H)­(BF_4_)] in water has two bands in the UV region (229 and 275 nm) and a broad band in the visible region (370 nm, Figure 3a, green). Titration with a calcium hydroxide solution shows that the complex is transformed into a new species. In particular, with the increasing Ca­(OH)_2_ concentration, the absorbance of the bands at 228 and 275 nm decreases, and the bands become broader and shift to red. In addition, a new band at 256 nm appears (Figure 3).

**Figure 3:**
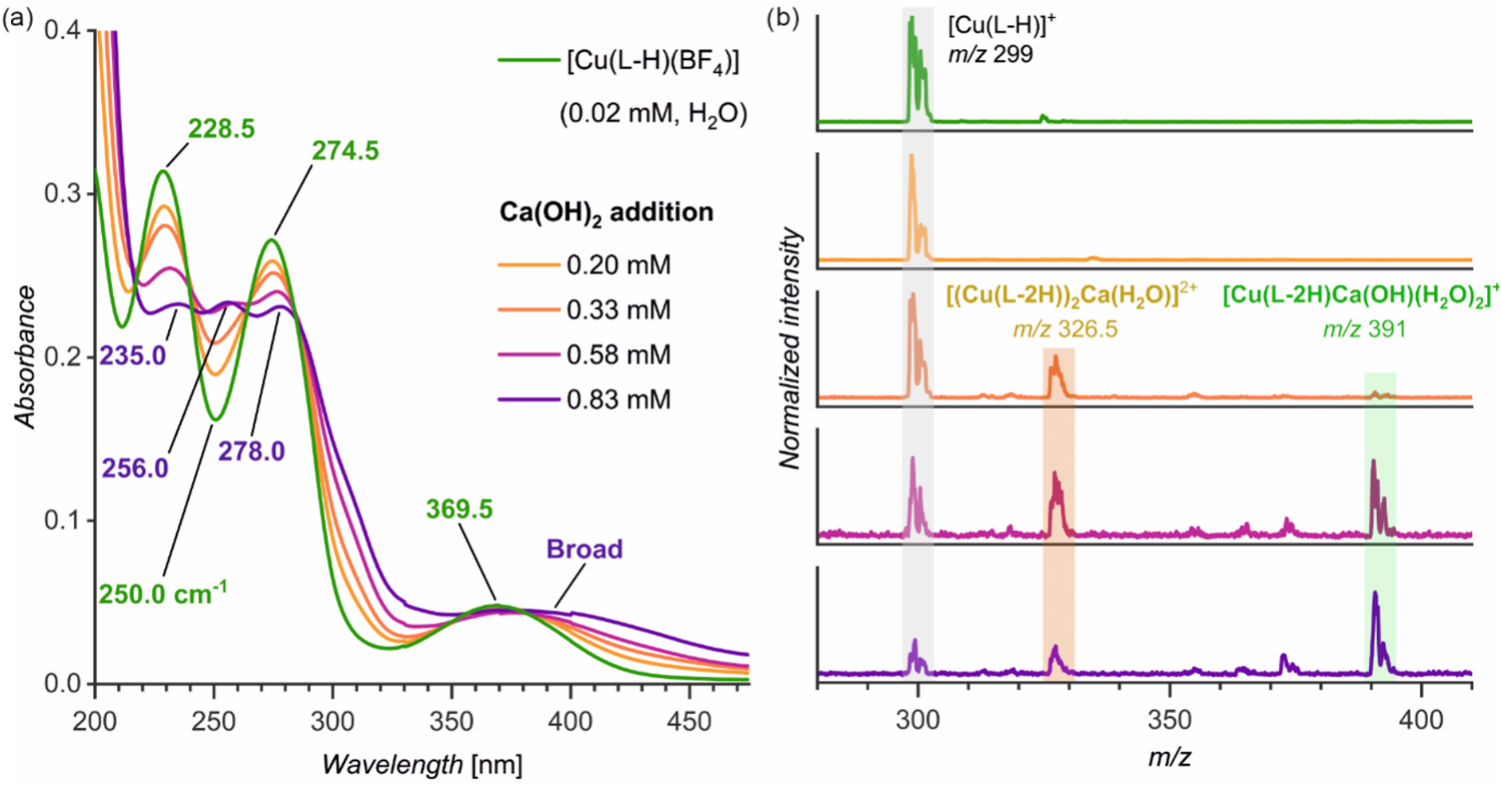
UV–vis (a) and ESI-MS (b) spectra of the aqueous solution of [Cu­(L-H)­(BF_4_)] and its titration with calcium hydroxide (experimental details are in the Supporting Information).

To unravel the changes in the speciation of the copper complex induced by adding Ca­(OH)_2_, we monitored the same solutions with electrospray ionization mass spectrometry (ESI-MS, see Figure S1 for high-resolution data). The analysis of the aqueous [Cu­(L-H)­(BF_4_)] shows the dominant signal of [Cu­(L-H)]^+^ (*m*/*z* 299), as expected (Figure 3b, green; see also Figure S2). Upon adding Ca­(OH)_2_, we detected calcium-bound dimer complexes with an additional water molecule, [(Cu­(L-2H))_2_Ca­(H_2_O)]^2+^ (*m*/*z* 326.5). The ligand L is dideprotonated, indicating that the calcium ion coordinates to the catecholate site of the ligand. At high Ca­(OH)_2_ concentrations (>0.58 mM), we also detected a monomer bearing two additional molecules of water and a hydroxide counterion, [Cu­(L-2H)­Ca­(OH)­(H_2_O)_2_]^+^ (*m*/*z* 391).

The changes in the ESI-MS spectra correlate with the changes in the UV–vis spectra. Therefore, it is likely that the change in the complex speciation is due to the coordination of the calcium ion to the catecholate binding site of the ligand. The composition of the monomer complex [Cu­(L-2H)­Ca­(OH)­(H_2_O)_2_]^+^ suggests the binding of two water molecules and the hydroxide anion to the calcium ion. During the collision-induced dissociation (CID) of [Cu­(L-2H)­Ca­(OH)­(H_2_O)_2_]^+^, we observed a consecutive elimination of three water molecules (Figure 4). The elimination of the third molecule of water must be associated with a migration of a proton from the primary amine group to the hydroxide anion. Alternatively, the primary amine can be deprotonated in the complex, coordinating the calcium ion with three molecules of water (Figure 4). To study the molecular structure of [Cu­(L-2H)­Ca­(OH)­(H_2_O)_2_]^+^ in detail, we further investigated the ions by helium tagging IRPD spectroscopy and DFT calculations.

**Figure 4:**
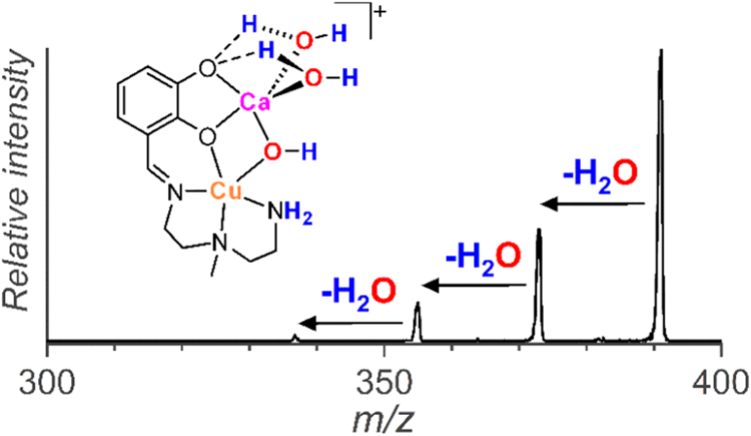
Collision-induced dissociation (CID) spectrum of [Cu­(L-2H)­Ca­(OH)­(H_2_O)_2_]^+^ (*m*/*z* 391).

### Infrared Photodissociation (IRPD) Spectra of the [Cu­(L-2H)­Ca­(OH)­(H_2_O)_2_]^+^ Complex

The structure of the [Cu­(L-2H)­Ca­(OH)­(H_2_O)_2_]^+^ complex is reflected in its infrared spectrum. For mass-selected ions, we can apply infrared photodissociation spectroscopy using helium tagging.^23–25^ The ions are trapped with helium buffer gas at 3.5 K, which leads to their internal relaxation to the ground vibrational state. Typically, the trapping and cooling also lead to rearrangements to the most stable isomers/conformers of the given mass-selected ions.^26^ If the isomers are close in energy and their population is expected based on thermodynamics, or the isomerization barriers are sufficiently high, higher energy isomers can also be studied.^27,28^

We measured the helium tagging IRPD spectrum of [Cu­(L-2H)­Ca­(OH)­(H_2_O)_2_]^+^ (Figure 5a, in black and Figure S4 in the Supporting Information). The sharp, intense absorption bands in the lower wavenumber range (1200–1400 cm^–1^) suggest that the complex is present most likely as a single isomer. These bands likely correspond to the C–O and C–N stretching modes. At higher wavenumbers, the IRPD spectrum shows rather broad bands. The absorption around 1450 cm^–1^ usually corresponds to methyl/methylene bending vibrations. The 1500–1650 cm^–1^ range is typical for double bond stretching, water bending, and primary amine bending modes. All these functionalities are represented in the investigated complex. Almost all bands are unusually broad, which is most likely due to hydrogen bonding of H_2_O molecules, which affects the flexible arms of the ligand. The broad absorption above 2800 cm^–1^ also suggests dynamic H_2_O binding. Two sharp peaks can be identified at 3413 cm^–1^ and 3732 cm^–1^, possibly corresponding to free N–H and O–H bond vibrations, respectively.

**Figure 5:**
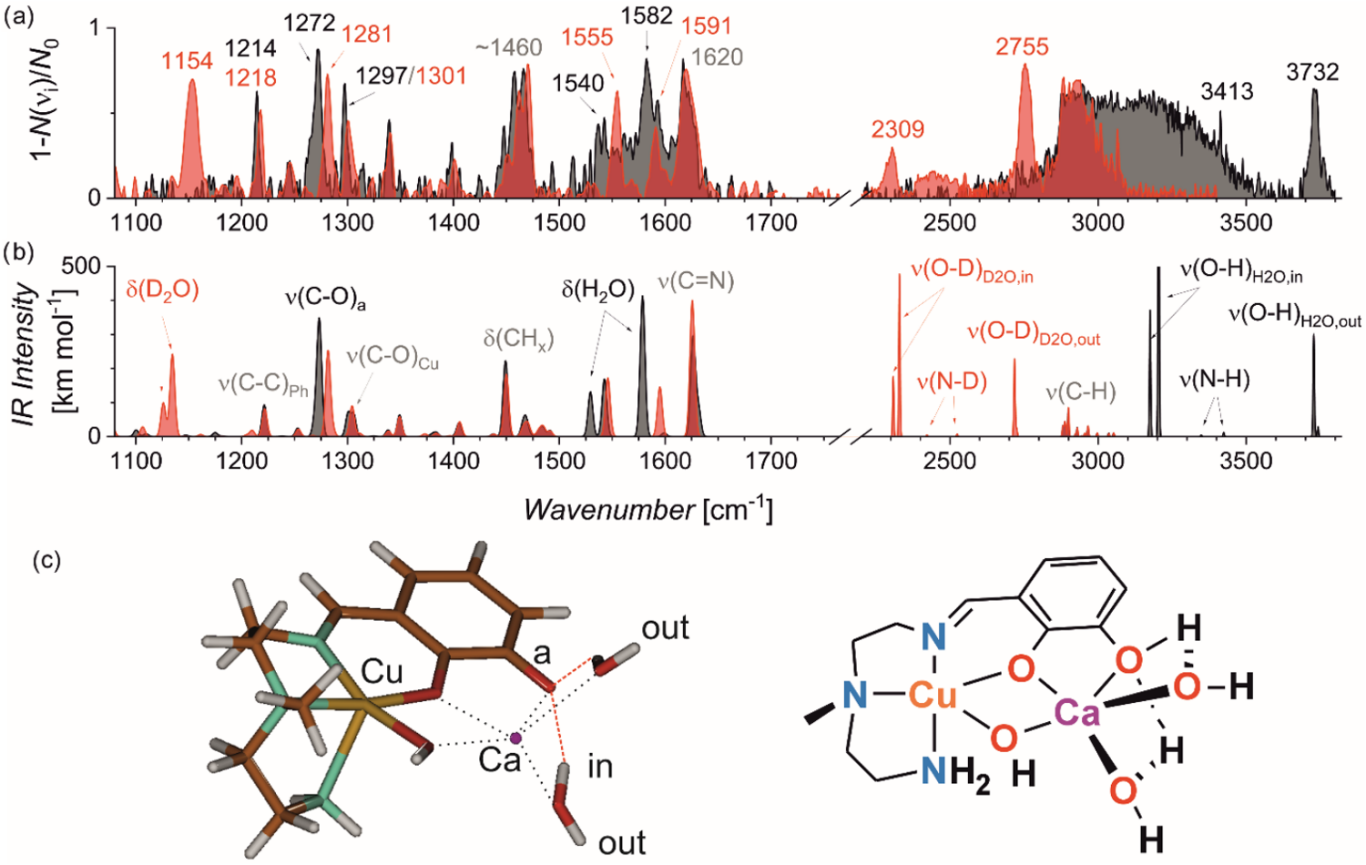
(a) Helium tagging IRPD spectra of [Cu­(L-2H)­Ca­(OH)­(H_2_O)_2_]^+^ (*m*/*z* 391, black) and D_7_-[Cu­(L-2H)­Ca­(OH)­(H_2_O)_2_]^+^ (*m*/*z* 398, red). (b) Theoretical IR spectra of the most stable isomer of [Cu­(L-2H)­Ca­(OH)­(H_2_O)_2_]^+^ and its D_7_-isotopolog in the respective colors (B3LYP-D3BJ/6–311++G**, scaling 0.985 and 0.955 below and above 2000 cm^–1^, respectively). (c) The optimized structure of the [Cu­(L-2H)­Ca­(OH)­(H_2_O)_2_]^+^ complex (left) and its symbolic drawing (right).

To analyze the effect of H_2_O binding on the IR spectrum, we measured the IRPD spectrum of the D_7_-deuterated analog of [Cu­(L-2H)­Ca­(OH)­(H_2_O)_2_]^+^. All acidic hydrogen atoms (N–H and O–H bonds) were exchanged with deuterium atoms (Figure S3). Upon this exchange, the broad absorption below 1600 cm^–1^ resolved to two bands at 1555 cm^–1^ and 1592 cm^–1^. Instead, we detected a broader band at 1154 cm^–1^ (Figure 5a, red spectrum). Another notable shift is observed for a band at 1272 cm^–1^, which blueshifts to 1281 cm^–1^.

Profound changes are found in the range of C–H, N–H, and O–H vibrations. Upon H/D exchange, the broad absorption covering the range from 3000 to 3500 cm^–1^ disappeared, meaning that the absorption belongs to the vibrational progression of the hydrogen-bonded O–H bonds. Instead, we detected bands at 2309 and 2755 cm^–1^ and broad absorptions in the 2380–2550 and 2850–3000 cm^–1^ ranges.

Knowing the spectroscopic signature of the complex, we investigated the organization of water molecules in the complex by DFT calculations. We optimized several structures with different positions of the water ligands and different sites of the ligand deprotonation. Nevertheless, most of the geometry optimizations led to one isomer that is 15 kJ mol^–1^ more stable than the next most stable structure (Figure 6). The favored isomer has square pyramidal O2N3 coordination of the copper ion by the imine and amine nitrogen atoms, one of the catecholate and the hydroxyl oxygen atoms in the plane, and the second amine atom in the apex. The calcium ion is coordinated in the plane to both catecholate oxygen atoms and the hydroxyl group. In addition, calcium ion binds two water molecules above and below the plane.

**Figure 6:**
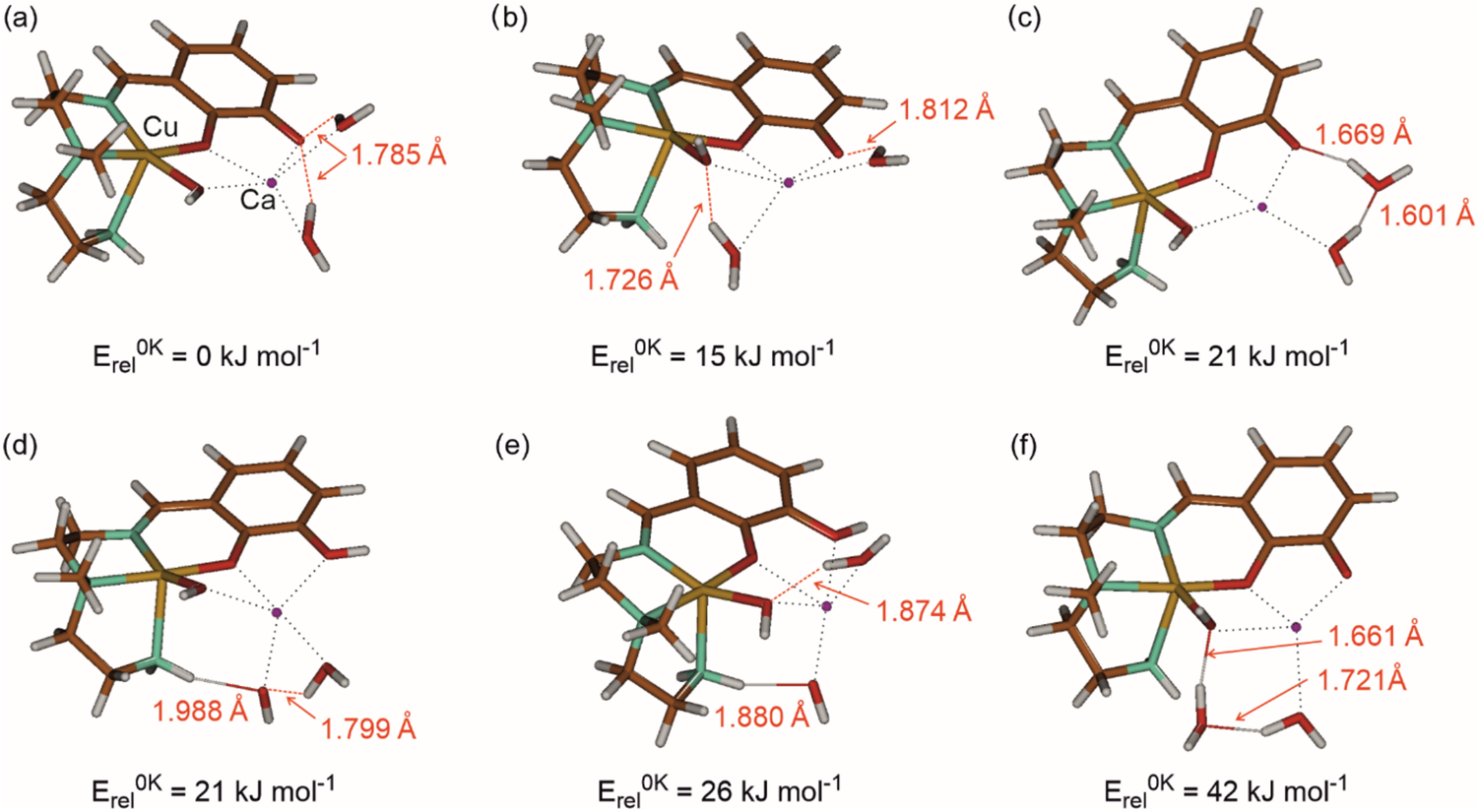
Optimized structures of different isomers of the [Cu­(L-2H)­Ca­(OH)­(H_2_O)_2_]^+^ complex (B3LYP-D3BJ/6–311++G**). The XYZ coordinates for visualization are provided in the Supporting Information. The distances are given for hydrogen bonding in the complex. Note that structures (d, e) contain a hydroxyl anion, while the ligand is only singly deprotonated.

The comparison of the most stable structure to less stable isomers shows that deprotonating the catechol ligand (structures a, b, c, f in Figure 6) is favored over deprotonating a water molecule (structures d and e). Further, the five-coordination of the calcium ion is energy-favored over the four-coordination (black dotted lines in Figure 6). All complexes have two hydrogen bonds (see the distances in Figure 6). The most stable isomer has two medium-length (1.785 Å) symmetric hydrogen bonds toward the catecholate oxygen atom.

The theoretical IR spectrum of the most stable complex (Figure 5b) agrees very well with the experimental IRPD spectrum. Analysis of the theoretical IR spectra allows us to assign the observed bands. The band at 1620 cm^–1^ is dominated by the imine C=N stretching vibration. The absorptions in the 1320–1500 cm^–1^ range are due to the deformation vibrations of methylene and methyl groups. The bands at 1540 and 1582 cm^–1^ in the H-isotopolog shift to the band at 1154 cm^–1^ in the D-isotopolog. These bands correspond to the H_2_O bending vibrations of the two water molecules coordinated with the calcium ion. The water molecules form a hydrogen bond with one of the catecholate oxygen atoms. This effect is also visible in the IR spectra as a blue shift of the corresponding C–O vibration at 1272 cm^–1^. Upon deuteration, this band shifts to 1281 cm^–1^.

The theoretical spectra reveal that the free O–H stretching band at 3732 cm^–1^ belongs to H_2_O molecules, to the stretching of their O–H bonds pointing outward the complex, not involving in hydrogen bonding. In the deuterated analog, the free O–D bonds vibrate at 2755 cm^–1^. The experimental isotopic shift of 977 cm^–1^ is slightly below that predicted based on Hooke’s law (1017 cm^–1^). The bonds involved in hydrogen bonding with the catecholate are theoretically predicted at 3177 and 3204 cm^–1^, but demonstrate a broad progression in the experimental spectrum. The IR signature of the O–D bonds engaged in hydrogen bonding with the catecholate oxygen atom is found at 2309 cm^–1^. The experimental O–D band is broad, covering both symmetric and antisymmetric vibrations of the hydrogen-bonded O–D bonds, but does not show the broad progression as found for the corresponding O–H vibration bands. Applying Hooke’s law, we can predict that the hydrogen-bonded O–H should be located at ∼3160 cm^–1^, which roughly coincides with the origin of the broad progression observed for the H-isotopolog. The N–H stretching vibration is detected at 3413 cm^–1^. Though the absorption intensity is predicted to be low, the absorption was detected reproducibly (see also Figure S4). In the deuterated analog, the N-D vibrations are in the range of a broad weak absorption around 2400 cm^–1^. Finally, the absorption above 2800 cm^–1^ that overlaps for both H- and D-isotopomers corresponds to the C–H stretching vibrations of the ligand.

The theoretical IR spectra of other localized structures can be found in the Supporting Information (Figure S5). Most spectra exhibit only minor differences but clearly fail to fit the experimental data as well as the spectrum of the most stable isomer.

## Discussion

This study shows that adding calcium hydroxide to the copper­(II) complex with a catecholate ligand, [Cu­(L-H)]^+^, leads to the formation of a copper–calcium complex [Cu­(L-2H)­Ca­(OH)­(H_2_O)_2_]^+^, where calcium binds to copper via a phenolate and hydroxide anions. Calcium ion is further stabilized by binding additional water molecules. This complex is stable; its formation was detected by electrospray ionization mass spectrometry and could be traced by changes in the UV–vis absorption of the aqueous solution of the copper complex during its titration with calcium hydroxide.

The water molecules coordinated to the calcium ion are further stabilized by hydrogen bonding with the ligand. The nature of the hydrogen bonding can be observed in the IRPD spectrum of the copper–calcium complex, [Cu­(L-2H)­Ca­(OH)­(H_2_O)_2_]^+^. The IRPD spectrum shows a band corresponding to free O–H bond vibrations, which belong to the O–H bonds of the water molecules pointing out from the complex. The inward-pointing O–H bonds interacting with the ligand reveal themselves as a broad progression. As studied earlier, such progression attests to dynamically bound water molecules, where stretching vibrations are coupled with the waging vibration, changing the bonding distance between the hydrogen bond and the hydrogen-bond acceptor.^29,30^ This progression is not present in the deuterated analog.

Concerning water oxidation catalysis, such precoordination might contribute to efficient reaction kinetics. Oxidation of a copper complex might lead to the formation of reactive copper-oxyl intermediates. The reactivity of the copper-oxyl unit is large and leads to many side reactions, notably the oxidative degradation of the organic ligands. Tight coordination of water molecules in the vicinity of the copper-oxyl moiety can lead to the favoring of the O–O bond formation and thus prevention of the undesired degradation reaction pathways. We did not study water oxidation using the complex reported here, because the ligand does not have optimum electronic properties. However, we will apply the principles learned in this study on how to achieve tight coordination of water in the vicinity of the copper reaction center to develop catalysts with optimized ligands in the future.

## Conclusions

We synthesized a copper complex with a redox-active ligand, [Cu­(L-H)­(BF_4_)], as a model system to study the water oxidation reaction. Here, we demonstrated that the complex transforms by adding calcium hydroxide into a stable copper–calcium complex, [Cu­(L-2H)­Ca­(OH)­(H_2_O)_2_]^+^, in which the calcium ion binds to the copper ions via bridging phenolate and hydroxide anions. Additionally, the calcium ion binds to two water molecules. The structural and spectroscopic characterization confirmed that the water molecules are stabilized through hydrogen bonding with the ligand. The helium-tagging IR photodissociation spectra, supported by DFT calculations, revealed signatures of free and hydrogen-bonded O–H vibrations, providing insights into the hydrogen-bonding environment. The coordination of water molecules around the reactive copper center mimics key features of biological water oxidation and suggests a strategy to enhance catalytic efficiency by promoting O–O bond formation while suppressing oxidative degradation pathways.

## Supplementary Material





## Notes

Experimental details (synthesis and characterization of [Cu­(L-H)­(BF_4_)], mass spectrometry and ion spectroscopy experimental details), additional mass spectrometry results, and results of DFT calculations (PDF)
DFT calculations XYZ output (XYZ)
