# Supplementary material for: Spectroscopic and Theoretical Investigation of Water Binding in a Copper–Calcium Complex
Source: J Phys Chem A. 2025 Jul 24;129(31):7140–5. doi: 10.1021/acs.jpca.5c03616 (PMC12337137; doi:10.1021/acs.jpca.5c03616)
Supplement: Supplementary file 1 [file jp5c03616_si_001.pdf]

Supporting Information

for

Spectroscopic and Theoretical Investigation of Water Binding in a Copper–Calcium Complex

*Noël de Kler, Aleksandr Y. Pereverzev, Jana Roithová**

Department of Spectroscopy and Catalysis, Institute for Molecules and Materials, Radboud University, 6525 AJ Nijmegen, The Netherlands. E-mail: jana.roithova@ru.nl

Contents

1. Materials and methods S3

1.1. Calcium hydroxide titration to [Cu(L-H)(BF_4)] S4

1.2. Preparation of [(Cu(L-H)(BF_4)] S4

1.3. Recrystallization of [(Cu(L-H)(BF_4)] – deposition number: 2314714 .... S4

1.4. XRD S5

1.5. Generation of ions with *m/z* 391 (TSQ) S7

1.6. Helium tagging IRPD spectroscopy S7

1.7. Density functional theory calculations S8

2. Experimental results S9

2.1. TIMS-TOF S9

2.2. ESI-MS (TSQ) S10

2.3. IRPD spectroscopy S11

2.4. DFT calculations S13

1. Materials and methods

Commercial solvents were used for the synthesis: Acetonitrile and methanol (VWR International). Calcium hydroxide, 2,2'-Diamino-N-methyldiethylamine and 2,3-dihydroxybenzaldehyde were bought from Sigma Aldrich. Solutions for ESI mass spectrometry were introduced into the instrument by a fused-silica capillary in solution with a slight overpressure. ESI-MS experiments for the calcium hydroxide titration experiments were performed with a triple-quadrupole instrument TSQ 7000 (Thermo) equipped with an electrospray ionization (ESI) source. UV-vis spectra were recorded on a JASCO V630 UV-Vis spectrophotometer. High-resolution mass spectrometry experiments were performed on a timsToF instrument (Bruker, Germany) with an ESI source. The ions were sprayed in positive mode with soft ionization conditions. Helium tagging IRPD experiments were recorded using the ISORI instrument. The calculated structures were optimized on with the B3LYP functional and 6-311++G** basis set and GD3BJ empirical dispersion correction, as implemented in the Gaussian 16 program. All reported structures are minima (no imaginary second derivative). The relative energies refer to 0 Kelvin and are expressed in kJ/mol.

1.1. Calcium hydroxide titration to [Cu(L-H)(BF_4)]

A stock solution of the complex [Cu(L-H)(BF_4)] (386 g/mol, 3.86 mg/ml, 10 mM) and calcium hydroxide (20 mM) were prepared in miliQ or D_2O (from Sigma Aldrich) and used for mass spectrometry and UV-vis experiments. Calcium hydroxide has poor solubility; therefore, the solution was sonicated before use until it formed a homogenous suspension. For mass spectrometry experiments, the sample was prepared in a 4 mL glass vial containing a septum. A mass spectrum was recorded of [Cu(L-H)(BF_4)] (0.02 mM) in water (3 mL). Next, calcium hydroxide (total volume: 30, 50, 90, and 130 uL, 20 mM) was added to the vial. After each addition, the solution was mixed by hand, and a mass spectrum was recorded.

UV-vis experiments were performed in a quartz cuvette containing 2.995 mL miliQ water to record a blank. Next, 5 uL [Cu(L-H)(BF_4)] (10 mM in H_2O) was added to the cuvette (final concentration 0.02 mM) and mixed with a Pasteur pipette, followed by the acquisition of the UV-vis spectrum. To the same solution, calcium hydroxide (20 mM) was added (total volume: 30, 50, 90 and 130 uL). After each addition the solution was mixed with a Pasteur pipette, and a UV-vis spectrum was recorded.

1.2.Preparation of [(Cu(L-H)(BF_4)]

[Cu(BF_4)$_2$] hexahydrate (202 mg, 0.852 mmol, 1eq) was dissolved in a mixture of methanol/acetonitrile (1:1, 8 mL). A solution of 2,2'-Diamino-N-methyldiethylamine (110 uL, 0.852 mmol) in 4 mL methanol was slowly added to the copper solution while stirring. After the addition, the solution was stirred for ~5 min, followed by adding 2,3-dihydroxybenzaldehyde (118 mg, 0.852 mmol) in 2 mL methanol while stirring. After stirring the solution for 3 hours, diethyl ether (~20 mL) was added, resulting in the complex's precipitation. The solution was filtered via vacuum filtration. The solids on the filter were washed with diethyl ether and dried to obtain a red crystalline material (170 mg, 52% yield). ESI-MS (H_2O), *m/z*: 299 ([(Cu(L-H)]$^+$), 685 ([(Cu(L-H))$_2$(BF_4)]$^+$).

1.3.Recrystallization of [(Cu(L-H)(BF_4)] – deposition number: 2314714

Single crystals were grown from a solution of [(Cu(L-H)(BF_4)] in methanol layered with diethyl ether. Slow diffusion of diethyl in methanol resulted in the formation of red crystals, which were characterized by X-ray diffraction.

1.4. XRD

Reflections were measured on a Bruker D8 Quest diffractometer with a sealed tube and Triumph monochromator (λ = 0.71073Å). The software package used for the intensity integration was Saint (v8.40a). Absorption correction was performed with SADABS. The structures were solved using direct methods using SHELXT-2014/5. Least-squares refinement was performed with SHELXL-2018/3 against $|F_h^o|^2$ of all reflections. Non-hydrogen atoms were refined freely with anisotropic displacement parameters. Hydrogen atoms were placed on calculated positions or located in difference Fourier maps. All calculated hydrogen atoms were refined with a riding model.

Crystal structure and structure refinement (2314714)

**General information**	
Identification code RU	NK220404s1- p2225a
Crystal colour	red
Crystal dimensions [mm] / shape	0.04 x 0.05 x 0.54 / needle
Crystallization solvent	MeOH/diethylether
Empirical formula	$C_{12}H_{18}CuN_3O_2$, BF_4
Formula weight [g/mol]	386.64
**Crystal Data**	
Crystal system	Monoclinic
Space group	$P2_1/c$ (#14)
Unit cell dimensions	
a, b, c [Å]	7.8795(4), 17.6581(11), 21.6929(11)
α, β, γ [°]	90, 90.727(4), 90
Volume [$Å^3$]	3018.0(3)
Z	8
Density (calculated) [g/cm^3]	1.702
Absorption coefficient (MoKα) [mm^{-1}]	1.502
F(000)	1576
**Data Collection**	
Temperature during experiment [K]	150
Wavelength [Å]	0.71073
θ Min-Max [°]	2.6, 28.4
Index range	$-10 \leq h \leq 10$; $-23 \leq k \leq 23$; $-28 \leq l \leq 28$
Tot., Uniq. Data, R(int)	50019, 7531, 0.032
Observed Data [I > 2.0 σ(I)]	6604
**Refinement**	
Nref, Npar	7531, 443
R, wR2, S	0.0340, 0.0798, 1.03
Min. and Max. Resd. Dens. [e/ $Å^3$]	-1.10, 1.11

1.5. Generation of ions with *m/z* 391 (TSQ)

For an optimal signal of *m/z* 391, the calcium hydroxide and [Cu(L-H)(BF_4)] complex were mixed prior to injection in the ESI mass spectrometer. After mixing the solutions, they were transferred through a ~20 μL reactor and into the mass spectrometer via a silica capillary. A solution of calcium hydroxide (1.33 mM) and [Cu(L-H)(BF_4)] (0.1 mM) was filled in a HSW Plastipak 3 mL syringe. LABM8 Syringe Pumps were used to transfer the solutions via a PEEK mixing tee to the mass spectrometer. The flow rate of the [Cu(L-H)(BF_4)] solution was 4 uL/min, and for the $Ca(OH)_2$ solution was 8 uL/min. The same procedure was used to generate the deuterated complex (*m/z* 398), but all solutions were prepared in D_2O instead of H_2O.

ESI-MS parameters (TSQ): Source voltage: 4.1 kV. Capillary temperature: 200 °C. Capillary voltage: 10 V. Tubelens: 110 V. Sheath gas: 40 psi.

1.6. Helium tagging IRPD spectroscopy

IRPD experiments were performed with the customized ISORI instrument. Scheme 1 shows the principal event sequence of the experiment. The instrument is equipped with an electrospray ionization (ESI) source and has a quadrupole (Q1) – quadrupole bender (QB) – octupole (O) – quadrupole ion trap (QIT) -quadrupole (Q2) geometry. The ions generated by ESI are mass-selected by Q1 and guided by QB and O to the quadrupole ion trap operating at ~3.5 K. The ions are trapped and thermalized by collisions with helium buffer gas. Helium is injected by a piezo valve in several 0.2 ms pulses separated by a 20 ms delay for a time of 200 ms. The thermalized ions form complexes with helium atoms. The ions are then ejected from the trap, and the number of helium-tagged ions is determined by mass analysis by Q2 and detected by a dynode–multiplier system operated in a counting mode. The experiment works with a 1 Hz frequency, and the trapped ions are irradiated in alternating cycles by a Nd/YAG laser-pumped tunable OPO/OPA system (Laser Vision). The number of helium-tagged ions is N_i and N_{i0} in the cycle with and without irradiation. The IRPD spectrum is constructed as ($1-N_i/N_{i0}$).

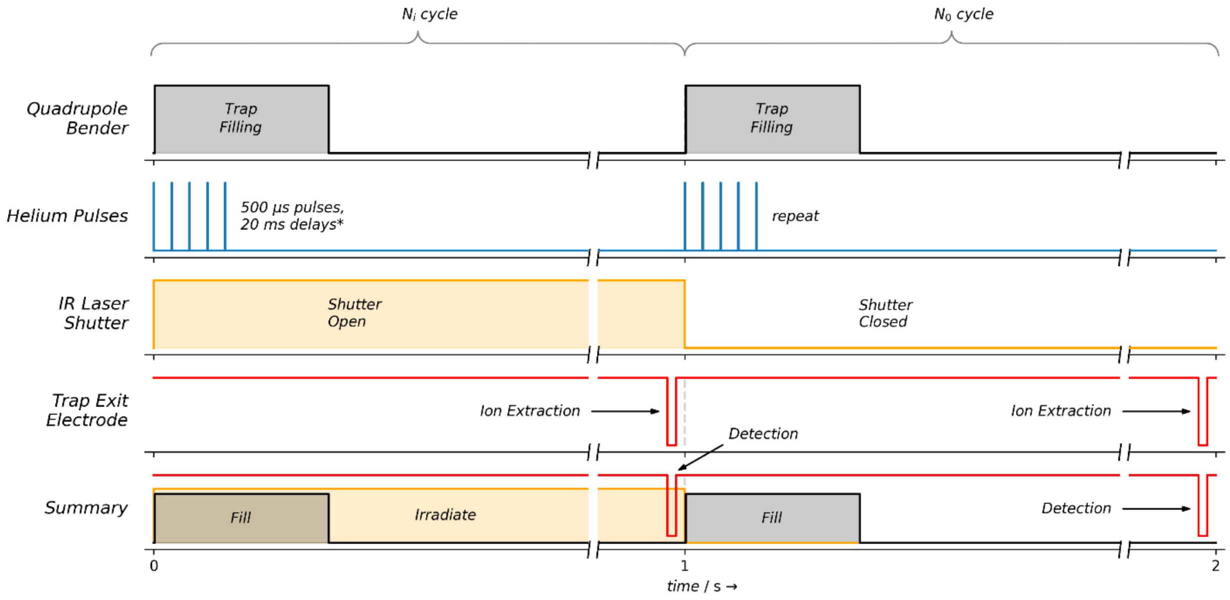

Scheme S1. Schematic representation of the pulse sequence used for IRPD.

1.7. Density functional theory calculations

The calculations were performed with the B3LYP functional using the D3BJ empirical correction for the dispersion interactions and the 6-311++G** basis set. All structures were fully optimized and confirmed by the frequency calculations. The reported theoretical IR spectra are harmonic and scaled by 0.985 below 2000 cm^{-1} and 0.955 above 2000 cm^{-1}. The calculations were performed with the Gaussian package.

2. Experimental results

2.1. TIMS-TOF

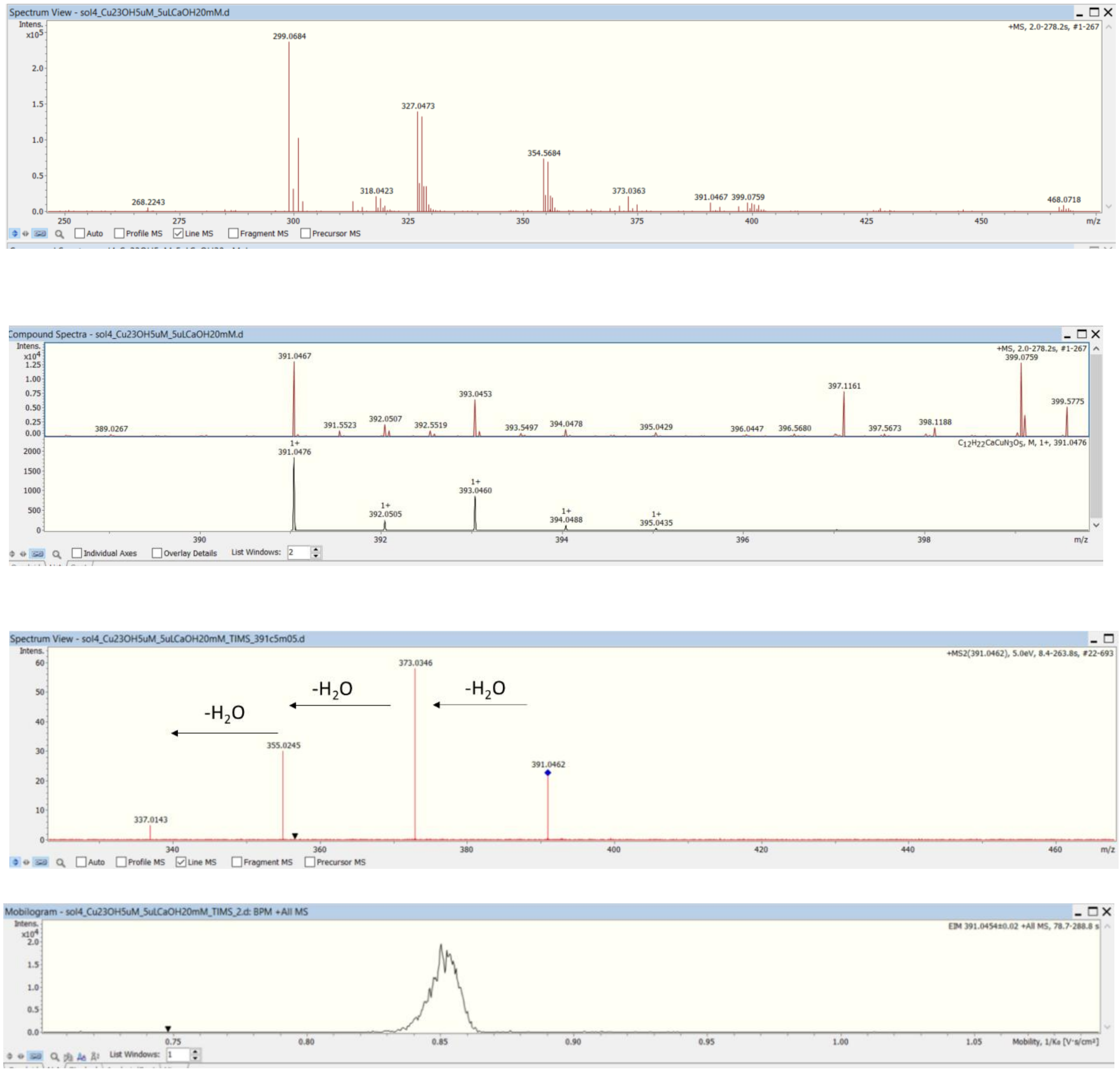

Figure S1. ESI-MS spectrum measured on TIMS-TOF. The theoretical isotopic pattern and accurate mass fits with the assignment $[Cu(L_{-2H})(CaOH(H_2O)_2]^{+}$. Collision induced dissociation of m/z 391 further confirms the assignment as a water cluster. Ion mobility shows that in TIMS-TOF only one isomer is detected.

2.2.ESI-MS (TSQ)

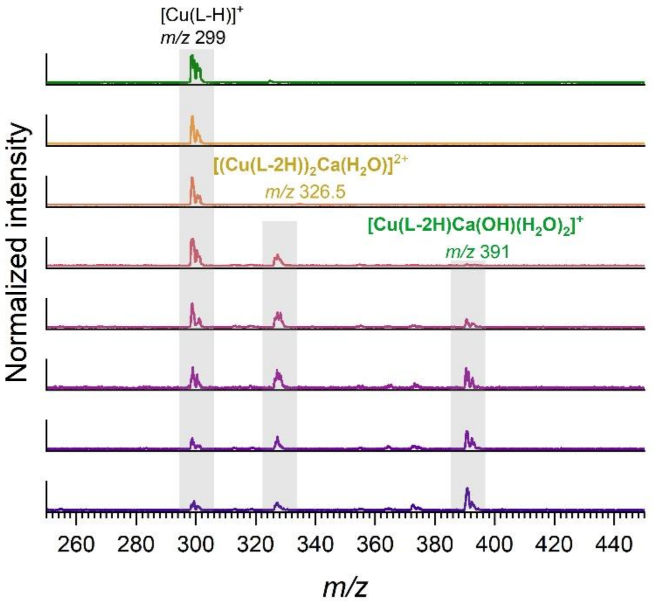

Figure S2. ESI-MS (TSQ) of calcium titration experiments. Yellow: [Cu(L-H)(BF_4)] (0.02 mM) and spectra after addition of calcium hydroxide (20 mM): 10, 30, 50, 70, 90, 110, 130 uL (increasing concentration from top to bottom).

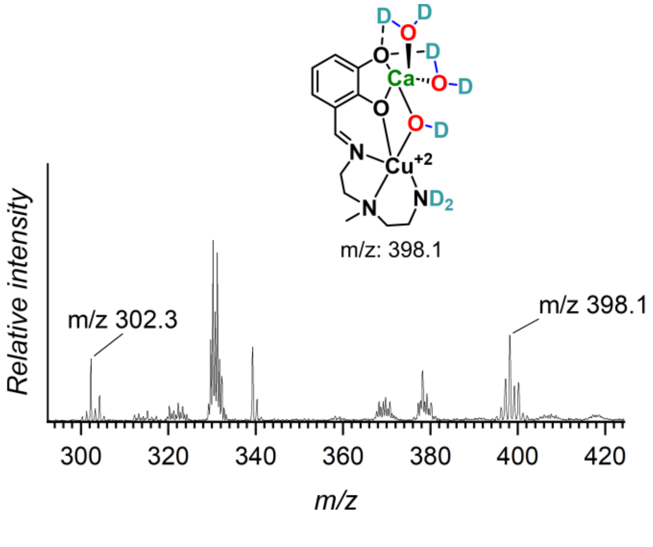

Figure S3. ESI-MS of [Cu(L-H)(BF_4)] (0.02 mM) and calcium hydroxide (0.8 mM) in D_2O showing the exchange of deuterium exchanged complex at m/z 398.1 (D7-[Cu(L-2H)Ca(OH)(H_2O)$_2$]$^+$.

2.3. IRPD spectroscopy

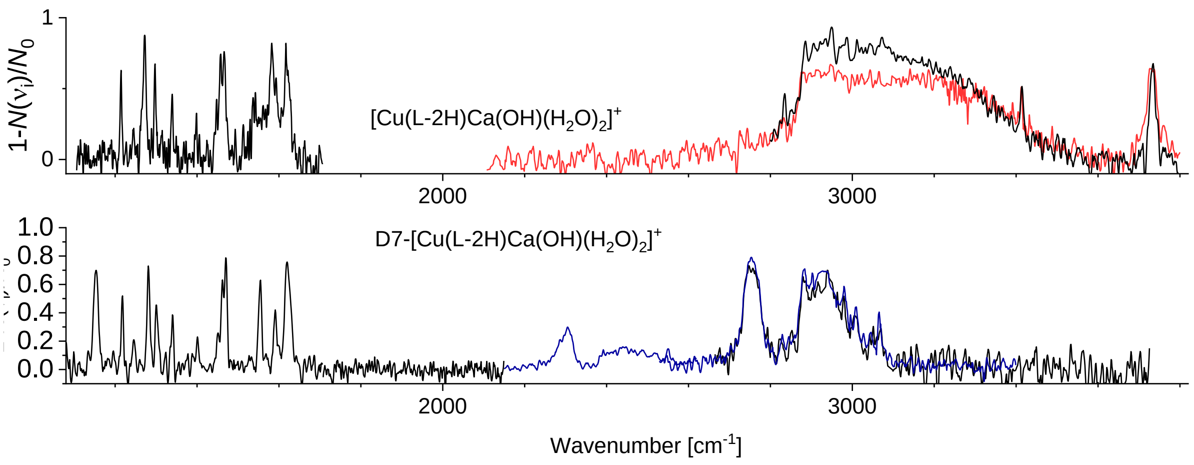

Figure S4. IRPD spectra of [Cu(L-2H)Ca(OH)(H_2O)$_2$]+ (m/z 391), a) and D7-[Cu(L-2H)Ca(OH)(H_2O)$_2$]+ (m/z 398, b). The colors show repeated experiments.

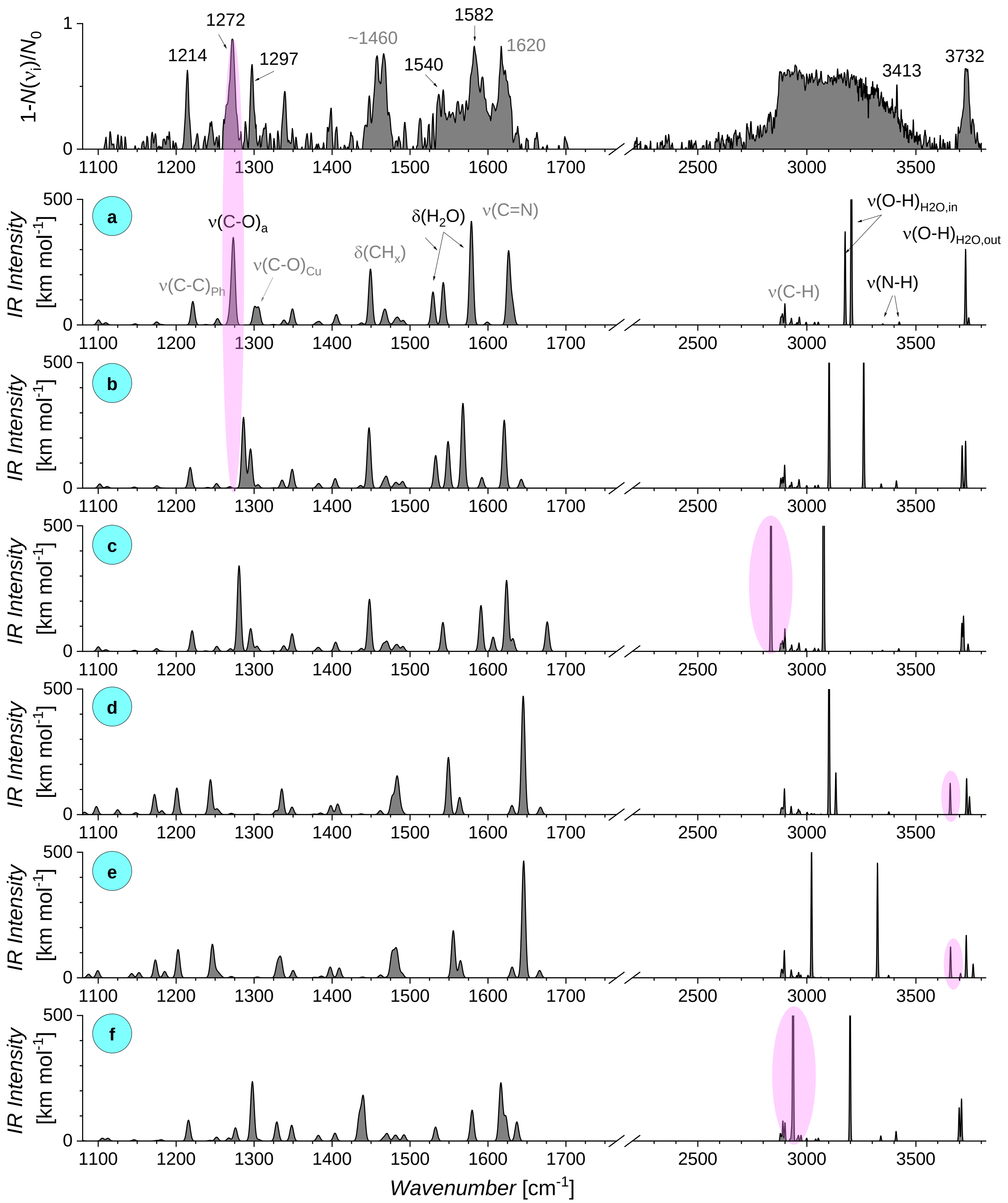

Figure S5. Comparison of the IRPD spectrum of $[Cu(L\text{-}2H)Ca(OH)(H_2O)_2]^+$ (*m/z* 391) with theoretical spectra is different isomers of the complex (Figure 6 in the main document, a-f notation is analogous to Figure 6). The pink ovals highlight the main disagreement between the experimental spectrum of the theoretical spectra of higher-energy isomers of $[Cu(L\text{-}2H)Ca(OH)(H_2O)_2]^+$.

2.4. DFT calculations

Energetics of the calculated structures

a

#N Geom=AllCheck Guess=TCheck SCRF=Check GenChk UB3LYP/6-311++G(d,p) Freq

Charge = 1 Multiplicity = 2

Zero-point correction= 0.352423 (Hartree/Particle)
Thermal correction to Energy= 0.378225
Thermal correction to Enthalpy= 0.379170
Thermal correction to Gibbs Free Energy= 0.297549
Sum of electronic and zero-point Energies= -3329.137933
Sum of electronic and thermal Energies= -3329.112130
Sum of electronic and thermal Enthalpies= -3329.111186
Sum of electronic and thermal Free Energies= -3329.192807

Low frequencies --- -2.0006 -0.0043 0.0045 0.0052 3.1034 4.4240
Low frequencies --- 25.8735 34.4526 59.1887

D7-a

#B3LYP/6-311+G** freq=noraman Empiricaldispersion=GD3BJ

Charge = 1 Multiplicity = 2

Zero-point correction= 0.329473 (Hartree/Particle)
Thermal correction to Energy= 0.356501
Thermal correction to Enthalpy= 0.357445
Thermal correction to Gibbs Free Energy= 0.273199
Sum of electronic and zero-point Energies= -3329.160519
Sum of electronic and thermal Energies= -3329.133491
Sum of electronic and thermal Enthalpies= -3329.132546
Sum of electronic and thermal Free Energies= -3329.216793

Low frequencies --- -1.9939 -0.0032 -0.0029 0.0030 3.1357 4.3375
Low frequencies --- 25.4196 33.0835 57.0107

b

#N Geom=AllCheck Guess=TCheck SCRF=Check GenChk UB3LYP/6-311++G(d,p) Freq

Charge = 1 Multiplicity = 2

Zero-point correction= 0.353009 (Hartree/Particle)
Thermal correction to Energy= 0.378468
Thermal correction to Enthalpy= 0.379412

Thermal correction to Gibbs Free Energy= 0.298436
Sum of electronic and zero-point Energies= -3329.132241
Sum of electronic and thermal Energies= -3329.106782
Sum of electronic and thermal Enthalpies= -3329.105838
Sum of electronic and thermal Free Energies= -3329.186814

Low frequencies --- -1.7962 -0.9877 0.0047 0.0050 0.0061 5.6782
Low frequencies --- 25.0625 28.8331 48.5695

c

#N Geom=AllCheck Guess=TCheck SCRF=Check GenChk UB3LYP/6-311++G(d,p) Freq

Charge = 1 Multiplicity = 2

Zero-point correction= 0.352726 (Hartree/Particle)
Thermal correction to Energy= 0.378301
Thermal correction to Enthalpy= 0.379245
Thermal correction to Gibbs Free Energy= 0.297115
Sum of electronic and zero-point Energies= -3329.129774
Sum of electronic and thermal Energies= -3329.104199
Sum of electronic and thermal Enthalpies= -3329.103254
Sum of electronic and thermal Free Energies= -3329.185385

Low frequencies --- 0.0026 0.0040 0.0048 1.7665 2.6374 3.6703
Low frequencies --- 18.4770 28.8815 46.1058

d

#N Geom=AllCheck Guess=TCheck SCRF=Check GenChk UB3LYP/6-311++G(d,p) Freq

Charge = 1 Multiplicity = 2

Zero-point correction= 0.352197 (Hartree/Particle)
Thermal correction to Energy= 0.378101
Thermal correction to Enthalpy= 0.379045
Thermal correction to Gibbs Free Energy= 0.296899
Sum of electronic and zero-point Energies= -3329.129971
Sum of electronic and thermal Energies= -3329.104067
Sum of electronic and thermal Enthalpies= -3329.103122
Sum of electronic and thermal Free Energies= -3329.185268

Low frequencies --- -0.8681 -0.0011 0.0055 0.0055 3.3742 5.6498
Low frequencies --- 14.8591 34.9270 43.4919

e

#N Geom=AllCheck Guess=TCheck SCRF=Check GenChk UB3LYP/6-311++G(d,p) Freq

Charge = 1 Multiplicity = 2

Zero-point correction= 0.352875 (Hartree/Particle)
Thermal correction to Energy= 0.378523
Thermal correction to Enthalpy= 0.379467
Thermal correction to Gibbs Free Energy= 0.298830
Sum of electronic and zero-point Energies= -3329.128026
Sum of electronic and thermal Energies= -3329.102379
Sum of electronic and thermal Enthalpies= -3329.101435
Sum of electronic and thermal Free Energies= -3329.182071

Low frequencies --- -3.7039 -0.0039 -0.0025 -0.0011 3.6044 5.6076
Low frequencies --- 23.6487 41.9350 64.1708

f

#N Geom=AllCheck Guess=TCheck SCRF=Check GenChk UB3LYP/6-311++G(d,p) Freq

Charge = 1 Multiplicity = 2

Zero-point correction= 0.353410 (Hartree/Particle)
Thermal correction to Energy= 0.378661
Thermal correction to Enthalpy= 0.379605
Thermal correction to Gibbs Free Energy= 0.299093
Sum of electronic and zero-point Energies= -3329.122087
Sum of electronic and thermal Energies= -3329.096837
Sum of electronic and thermal Enthalpies= -3329.095893
Sum of electronic and thermal Free Energies= -3329.176405

XYZ coordinates are in a separate file for easier visualization.
